# Supplementary material for: The T-cell receptor repertoire of wild mice
Source: Discov Immunol. 2026 Feb 20;5(1):kyag002. doi: 10.1093/discim/kyag002 (PMC12994567; doi:10.1093/discim/kyag002)
Supplement: kyag002_Supplementary_Data [file kyag002_supplementary_data.docx]

**Supplementary Material 1.** Example FACS plots of a mouse splenocyte sample where (A) cells were stained with DAPI, CD3-PE, CD4-FITC, and CD8-APC and (B) where for the same sample DAPI, CD3-PE, CD4-FITC, and CD8-APC were omitted from the staining protocol. For each, the gating hierarchy are plotted sequentially as (i) FSC-A *vs*. SSC-A, (ii) FSC-A *vs.* FSC-H, (iii) DAPI *vs.* SSC, (iv) CD3-PE *vs.* SSC, (v) CD4-FITC *vs.* CD8-APC, and the subsequent CD3^+^ CD4^+^ and CD3^+^ CD8^+^ were defined from dot plots of pair-matched negative controls (no antibody staining) from each individual (B), where maximum tolerance to false positive was <0.3 %.

**A**


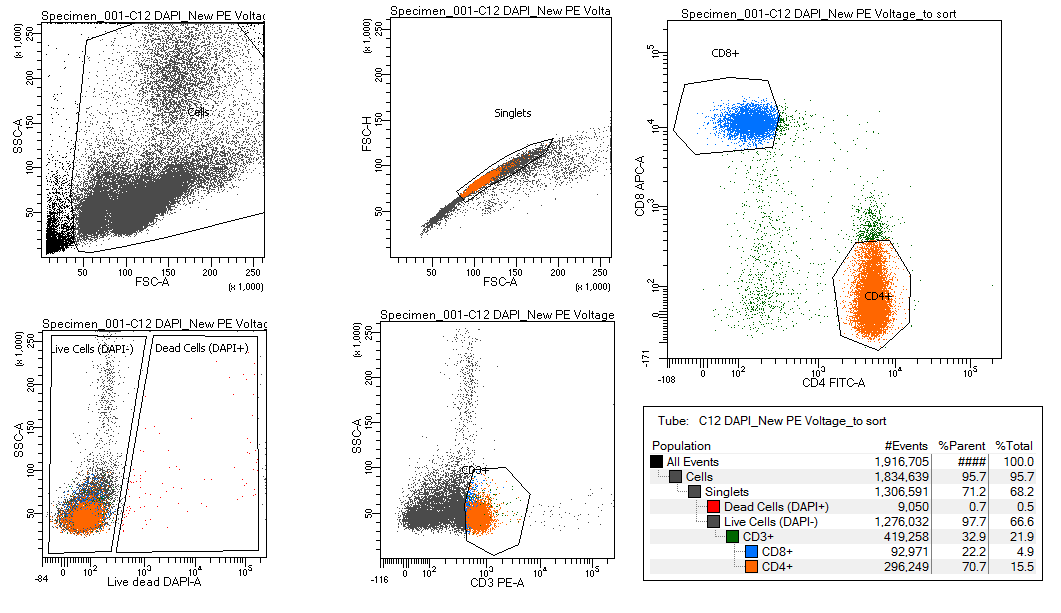


**B**


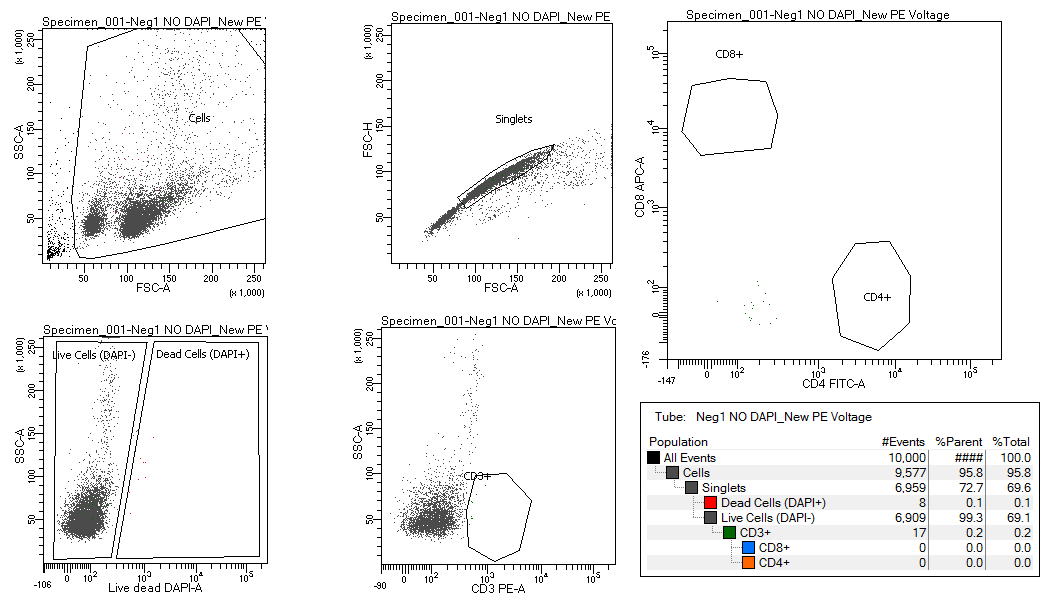


**Supplementary Material 2**. Mouse age in days and (A) spleen mass in grammes (Spleen mass = 0.03 + 0.0002.Age, R^2^ = 0.3, p < 0.0001) and (B) number of splenocytes (Number of splenocytes = 1.02 x 10^6^ + 6.37 x 10^4^.Age,R^2^ = 0.13, p < 0.0025).

**A**


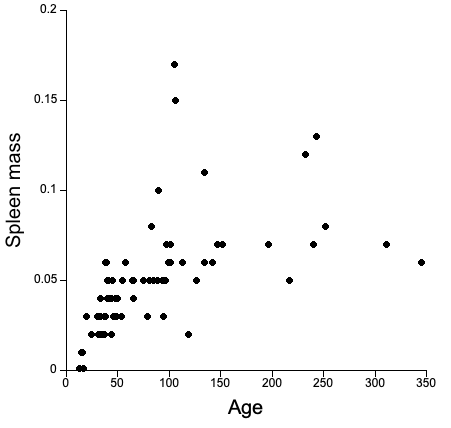


**B**


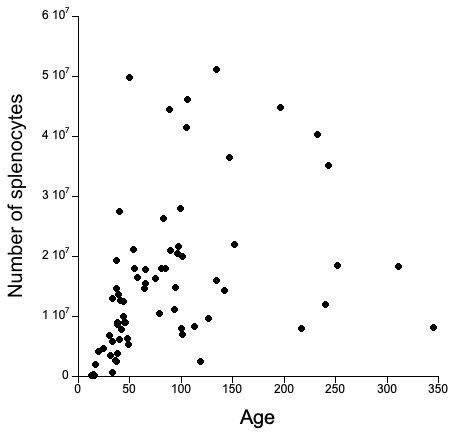


**Supplementary Material 3**. The power law coefficients of the frequency distribution of amino acid-defined TCR sequences for wild mice from the two sample sites for (A) CD4^+^ and (B) CD8^+^ cells; within each an example Log Frequency *vs.* Log Number of amino acid-defined sequences is shown. (C) Result of Kolmogorov-Smirnov test with 500 bootstraps to test that the data follows a power law distribution, showing the number of mice in each category.

**A**


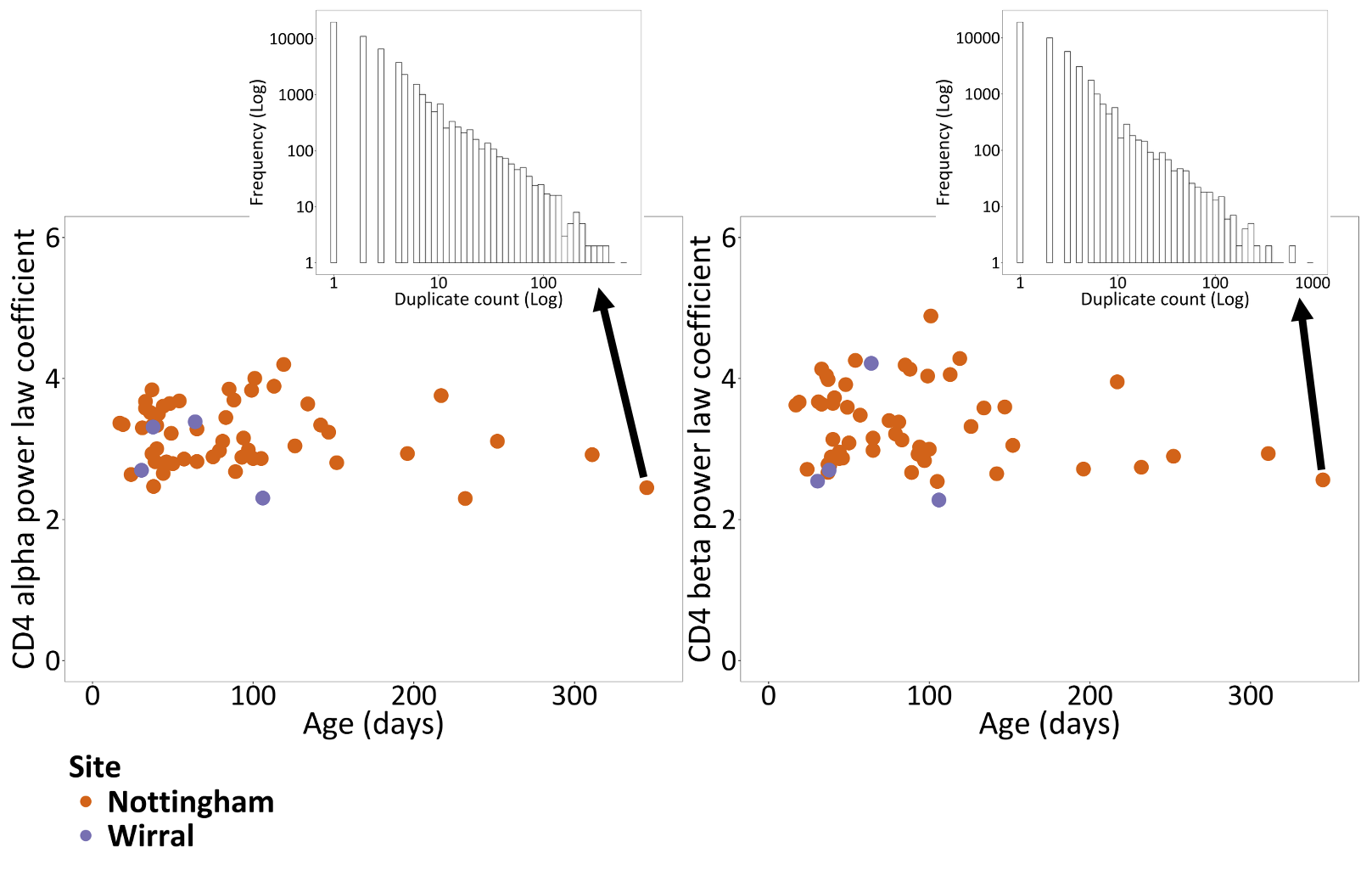


**B**

**
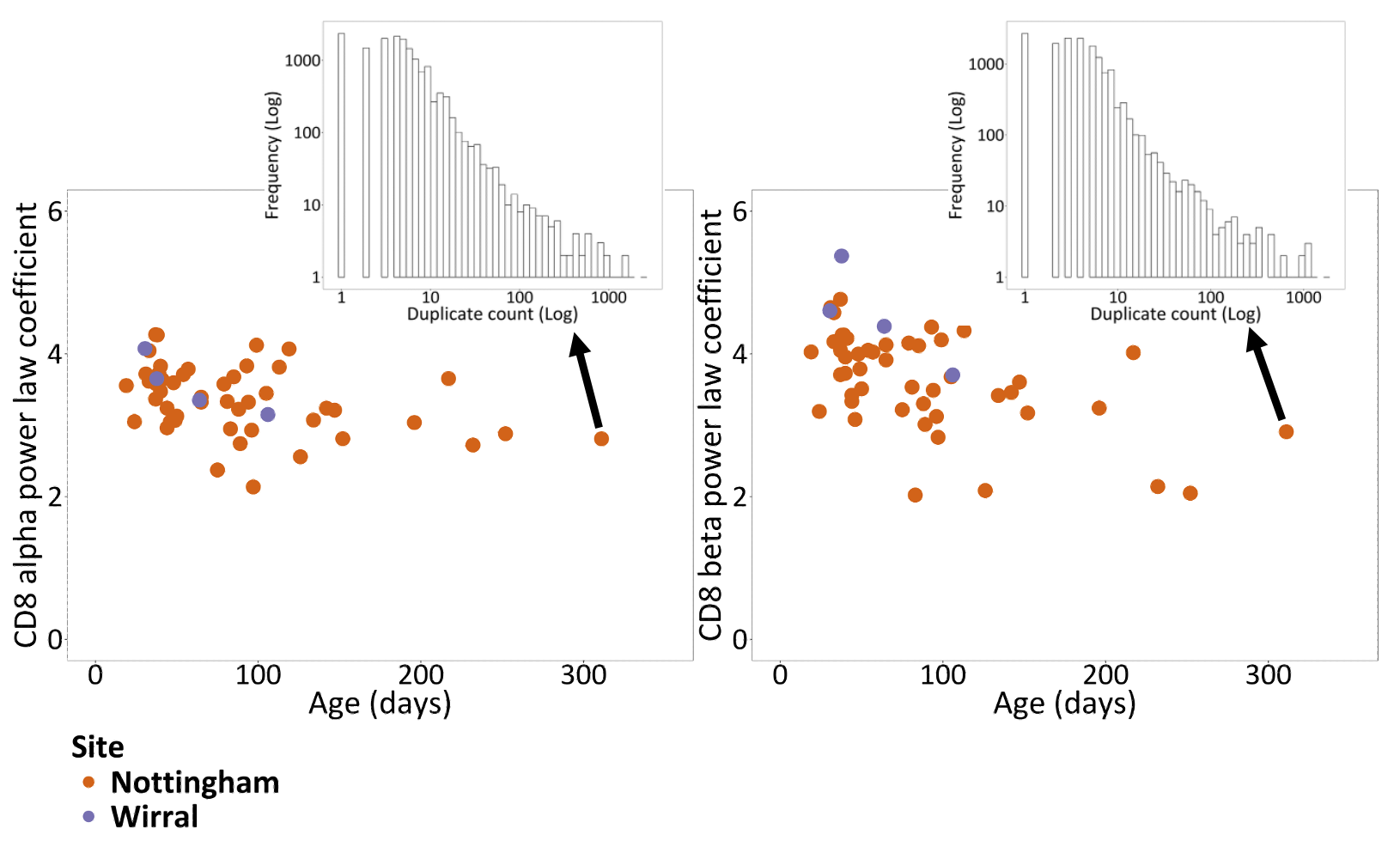
**

**C**

| **Receptor type** | **Fit to power law distribution** | | |
| --- | --- | --- | --- |
|  | **Fits (p > 0.1)** | **Borderline fit (0.05 < p < 0.1)** | **Does not fit (p < 0.05)** |
| **CD4^+^ alpha** | n = 45 (72.6%) | n = 3 (4.8%) | n = 14 (22.6%) |
| **CD4^+^ beta** | n = 24 (38.7%) | n = 3 (4.8%) | n = 35 (56.5%) |
| **CD8^+^ alpha** | n = 24 (41.4%) | n = 4 (6.9%) | n = 30 (51.7%) |
| **CD8^+^ beta** | n = 10 (17.2%) | n = 2 (3.4%) | n = 46 (79.3%) |

**Supplementary Material 4**. (A) Wild mouse TCR richness (calculated as the number of unique amino acid-defined TCRs as a proportion of the total number of amino acid-defined TCR sequences); the whiskers are 1.5 x the inter-quartile range; * is p < 0.05. (B) The effect sizes (top value) and p values (bottom value) for pairwise comparisons of the marginal means estimated from a GLM of the form VALUE ~ RECEPTOR TYPE + SAMPLE SITE + AGE + SEX; p < 0.05 are in bold. (C) Summary of wild mouse TCR richness GLM. Estimates are shown relative to reference levels (CD4^+^ alpha, Nottingham, Female). Coefficients with p < 0.05 are in bold. GLM details were: n = 216; residual df = 208; null deviance = 37.40; residual deviance = 33.61; AIC = −495.5.

**A**


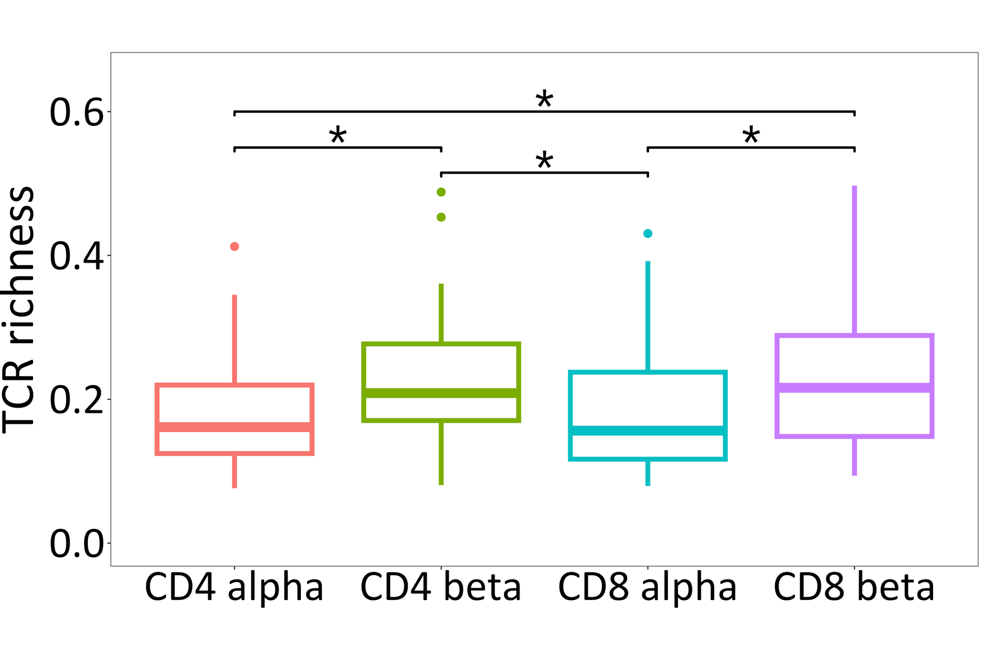


**B**

|  | **CD4^+^ alpha** | **CD4^+^ beta** | **CD8^+^ alpha** |
| --- | --- | --- | --- |
| **CD4^+^ beta** | **1.243**  **0.011** |  |  |
| **CD8^+^ alpha** | 0.053  0.9994 | **-1.191**  **0.0197** |  |
| **CD8^+^ beta** | **1.211**  **0.0168** | -0.032  0.9997 | **1.159**  **0.0281** |

**C**

| **Coefficient** | **Estimate** | **SE** | **z** | **p** |
| --- | --- | --- | --- | --- |
| **(Intercept)** | **5.999** | **0.432** | **13.89** | **<0.001** |
| Site (Wirral *vs.* Nottingham) | −0.198 | 0.519 | −0.38 | 0.704 |
| Age (days) | −0.00064 | 0.00259 | −0.25 | 0.804 |
| **CD4^+^ beta (*vs.* CD4^+^ alpha)** | **−1.243** | **0.399** | **−3.11** | **0.002** |
| CD8^+^ alpha (*vs.* CD4^+^ alpha) | −0.053 | 0.452 | −0.12 | 0.908 |
| **CD8^+^ beta (*vs.* CD4^+^ alpha)** | **−1.211** | **0.406** | **−2.98** | **0.003** |
| Sex (Male *vs.* Female) | −0.549 | 0.487 | −1.13 | 0.261 |
| Age*Sex | 0.00009 | 0.00469 | 0.02 | 0.985 |

**Supplementary Material 5**. (A) Wild mouse amino acid-defined TCR alpha diversity using Shannon’s index; the whiskers are 1.5 x the inter-quartile range; * is p < 0.05. (B) The effect sizes (top value) and p values (bottom value) for pairwise comparisons of the marginal means estimated from a GLM of the form VALUE ~ RECEPTOR TYPE + SAMPLE SITE + AGE + SEX + AGE*SEX ; p < 0.05 are in bold. (C) Summary of Shannon’s diversity index GLM. Estimates are shown relative to reference levels (CD4^+^ alpha, Nottingham, Female). Coefficients with p < 0.05 are in bold. GLM details were: n = 216; residual df = 208; null deviance = 43.23; residual deviance = 36.10; AIC = 244.5.

**A**


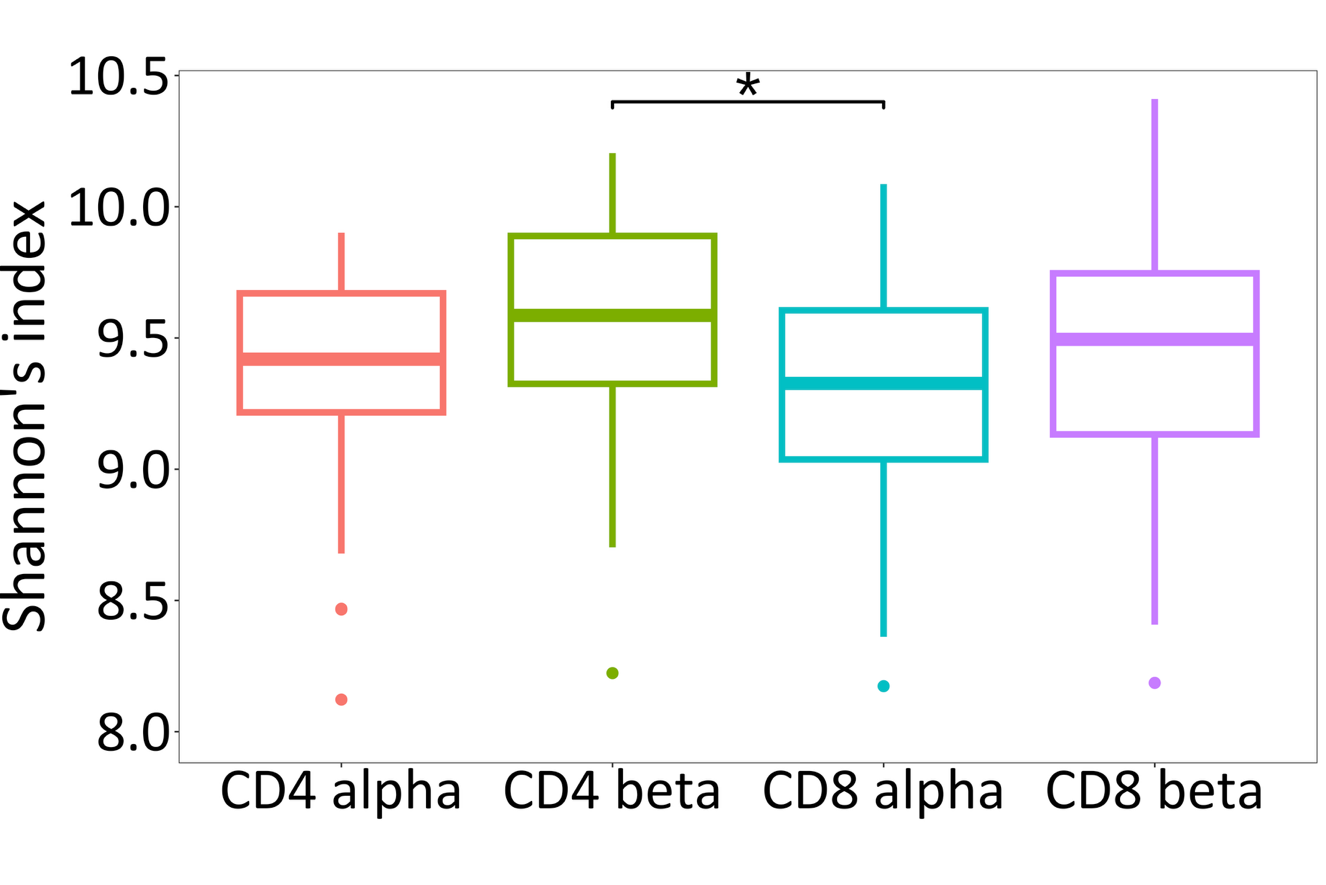


**B**

|  | **CD4^+^ alpha** | **CD4^+^ beta** | **CD8^+^ alpha** |
| --- | --- | --- | --- |
| **CD4^+^ beta** | -0.186  0.0876 |  |  |
| **CD8^+^ alpha** | 0.070  0.8197 | **0.256**  **0.0089** |  |
| **CD8^+^ beta** | -0.064  0.8576 | 0.122  0.4244 | -0.134  0.3614 |

**C**

| **Coefficient** | **Estimate** | **SE** | **t** | **p** |
| --- | --- | --- | --- | --- |
| **(Intercept)** | **9.375** | **0.080** | **117.82** | **<0.001** |
| **Site (Wirral *vs.* Nottingham)** | **0.303** | **0.110** | **2.76** | **0.006** |
| **Age (days)** | −0.00090 | 0.00051 | −1.78 | 0.076 |
| **CD4^+^ beta (*vs.* CD4^+^ alpha)** | **0.186** | **0.079** | **2.36** | **0.019** |
| CD8^+^ alpha (*vs.* CD4^+^ alpha) | −0.070 | 0.080 | −0.87 | 0.385 |
| CD8^+^ beta (*vs.* CD4^+^ alpha) | 0.064 | 0.080 | 0.79 | 0.429 |
| **Sex (Male *vs.* Female)** | **0.280** | **0.100** | **2.79** | **0.0058** |
| **Age*Sex** | **−0.00205** | **0.00099** | **−2.07** | **0.0396** |

**Supplementary Material 6**. (A) Wild mouse amino acid-defined TCR alpha diversity using Simpson’s index; the whiskers are 1.5 x the inter-quartile range; * is p < 0.05. (B) The effect sizes (top value) and p values (bottom value) for pairwise comparisons of the marginal means estimated from a GLM of the form VALUE ~ RECEPTOR TYPE + SAMPLE SITE + AGE + SEX + AGE*SEX ; p < 0.05 are in bold. (C) Summary of Simpson’s diversity index GLM. Estimates are shown relative to reference levels (CD4^+^ alpha, Nottingham, Female). Coefficients with p < 0.05 are in bold. GLM details were: n = 216; residual df = 208; null deviance = 166.8; residual deviance = 129.0; AIC = 519.6.

**A**


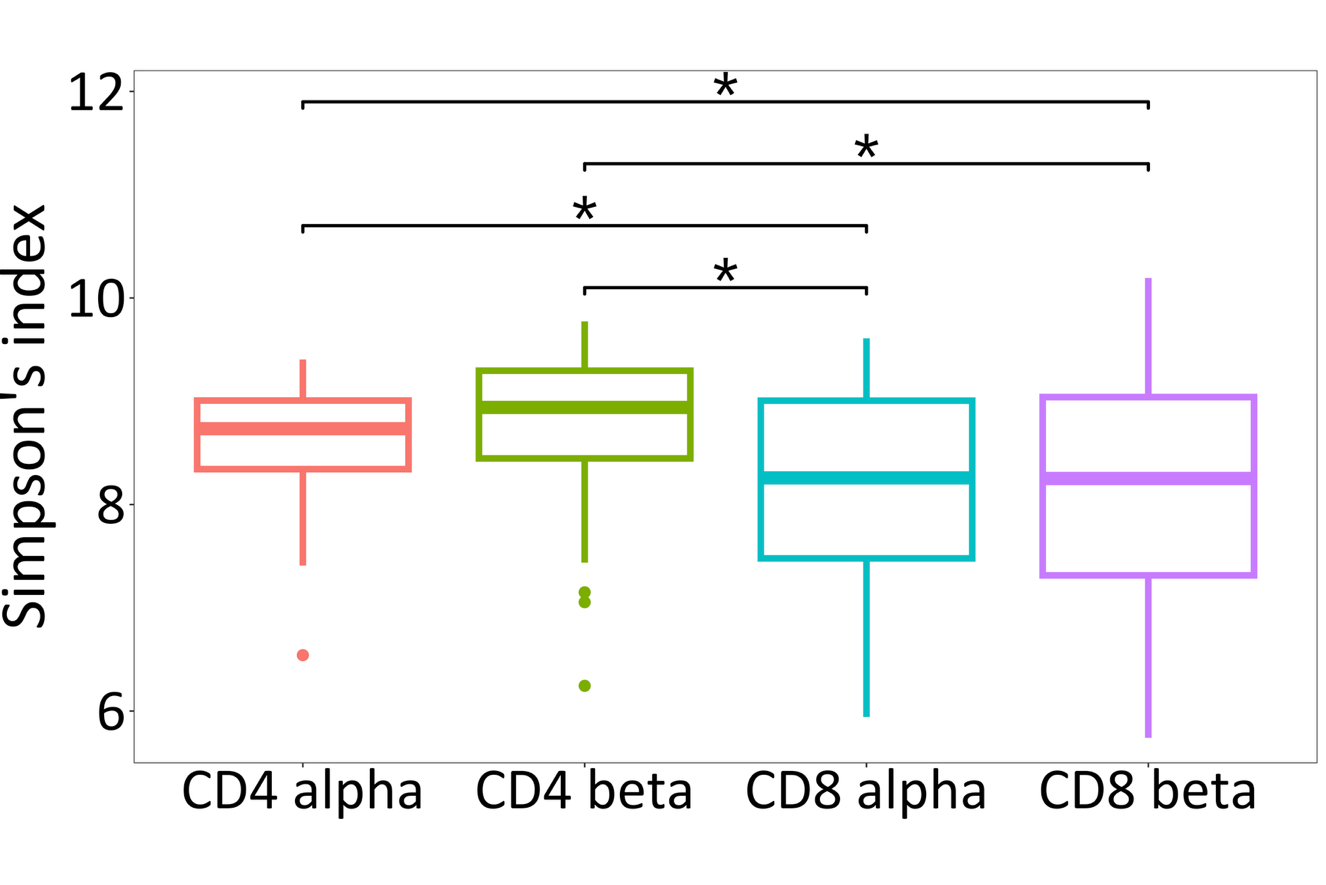


**B**

|  | **CD4^+^ alpha** | **CD4^+^ beta** | **CD8^+^ alpha** |
| --- | --- | --- | --- |
| **CD4^+^ beta** | -0.165  0.6853 |  |  |
| **CD8^+^ alpha** | **0.434**  **0.0240** | **0.599**  **0.0006** |  |
| **CD8^+^ beta** | **0.445**  **0.0195** | **0.610**  **0.0005** | 0.011  0.9999 |

**C**

| **Coefficient** | **Estimate** | **SE** | **t** | **p** |
| --- | --- | --- | --- | --- |
| **(Intercept)** | **8.861** | **0.150** | **58.91** | **<0.001** |
| Site (Wirral *vs.* Nottingham) | 0.030 | 0.207 | 0.15 | 0.884 |
| **Age (days)** | **−0.00380** | **0.00096** | **−3.97** | **<0.001** |
| CD4^+^ beta (*vs.* CD4^+^ alpha) | 0.165 | 0.149 | 1.11 | 0.269 |
| **CD8^+^ alpha (*vs.* CD4^+^ alpha)** | **−0.434** | **0.152** | **−2.86** | **0.0047** |
| CD8^+^ beta (*vs.* CD4^+^ alpha) | −0.445 | 0.152 | −2.93 | 0.0038 |
| **Sex (Male *vs.* Female)** | **0.383** | **0.190** | **2.02** | **0.045** |
| Age*Sex | −0.00222 | 0.00187 | −1.19 | 0.236 |

**Supplementary Material 7**. The results of GLM analysis of effects on wild mouse TCR diversity measured using Shannon and Simpson’s diversity indices, of the form VALUE ~ RECEPTOR TYPE + SITE + AGE + SEX + SEX*AGE, where for each term or the interaction the parameter estimate and the p values are shown. NS is not significant at p = 0.05.

|  | **Shannon’s** | **Simpson’s** |
| --- | --- | --- |
| **Age** | -0.00090, p = 0.0762 | -0.00380, p = 9.91x10^-5^ |
| **Sex (Male)** | 0.2803, p = 0.0058 | 0.3827, p = 0.0452 |
| **Sample site (Wirral)** | 0.30279, p = 0.0063 | NS |
| **Age*Sex (Male)** | -0.00205, p = 0.0396 | NS |

**Supplementary Material 8**. (A) The mean and median number of amino acid-defined TCR sequences shared among wild and laboratory mice and, in parentheses, these expressed as a percentage of the number of down-sampled sequences. (B) The number of amino-acid defined TCR sequences shared among >75 % of mice (both wild and laboratory) and this expressed as a percent of all the unique TCR sequences.

**A**

|  | **CD4^+^ alpha** | **CD4^+^ beta** | **CD8^+^ alpha** | **CD8^+^ beta** |
| --- | --- | --- | --- | --- |
| **Mean** | 3,232 (5.89) | 1,678 (2.86) | 2,561 (5.19) | 1,387 (2.16) |
| **Median** | 3,221 (5.87) | 1601 (2.72) | 2,459 (4.98) | 1,251 (1.95) |
| **Number of down-sampled sequences** | 54,908 | 58,772 | 49,379 | 64,224 |

**B**

| **Receptor type** | **Number** | **Percent** |
| --- | --- | --- |
| **CD4^+^ alpha** | 1,113 | 0.199 |
| **CD4^+^ beta** | 268 | 0.024 |
| **CD8^+^ alpha** | 703 | 0.143 |
| **CD8^+^ beta** | 157 | 0.017 |

**Supplementary Material 9**. Principal Co-ordinates Analysis (PCoA) showing the first two principal co-ordinates of the number of shared amino acid-defined TCR sequences between all pairwise combinations of wild and laboratory mice, with the origins of the mice colour coded for (A) CD4^+^ alpha, (B) CD4^+^ beta, (C) CD8^+^ alpha and (D) CD8^+^ beta. The percentage of variance explained by PCo1 and PCo2 is for CD4^+^ alpha 11.1 and 9,4; CD4^+^ beta 21.4 and 8.3; CD8^+^ alpha 10.3 and 8.4; CD8^+^ beta 15.5 and 7.5, respectively.

**A**


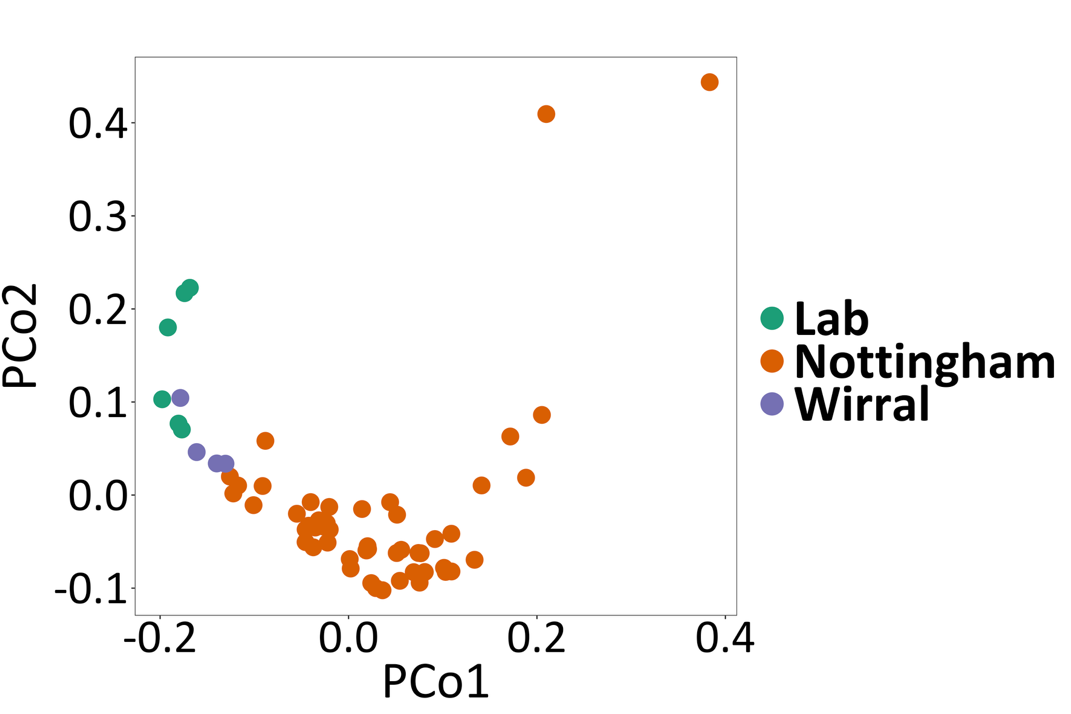


**B**


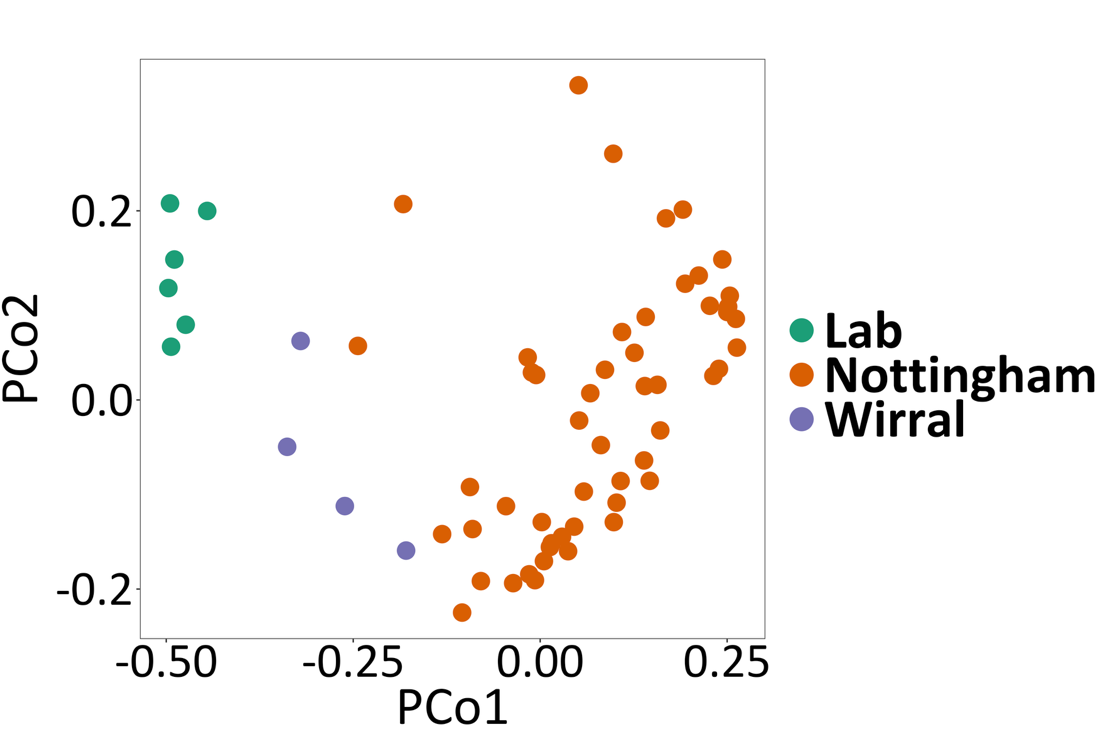


**C**


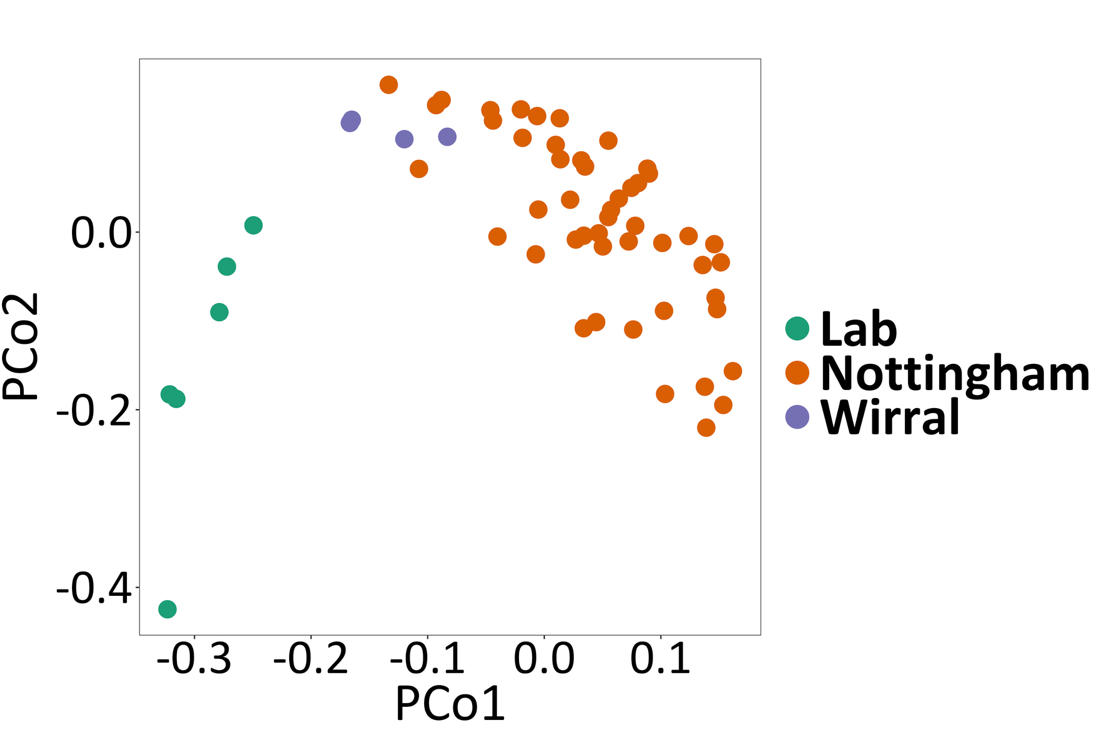


**D**


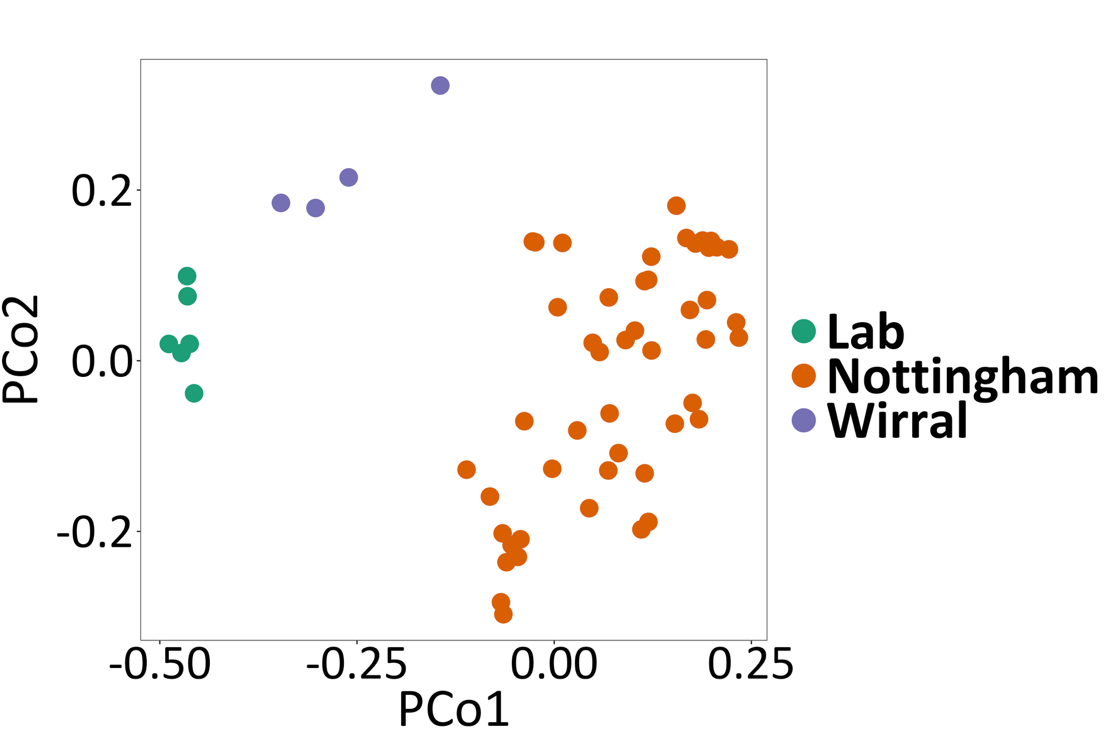


**Supplementary Material 10**. The number of shared amino acid-defined TCR sequences between all pairwise combinations of wild mice from the Nottingham site against the difference in pairwise age (left hand panels) and the sum of the pairwise age (right hand panels), where age is in days for (A) CD4^+^ alpha, (B) CD4^+^ beta and (C) CD8^+^ beta.

**A**


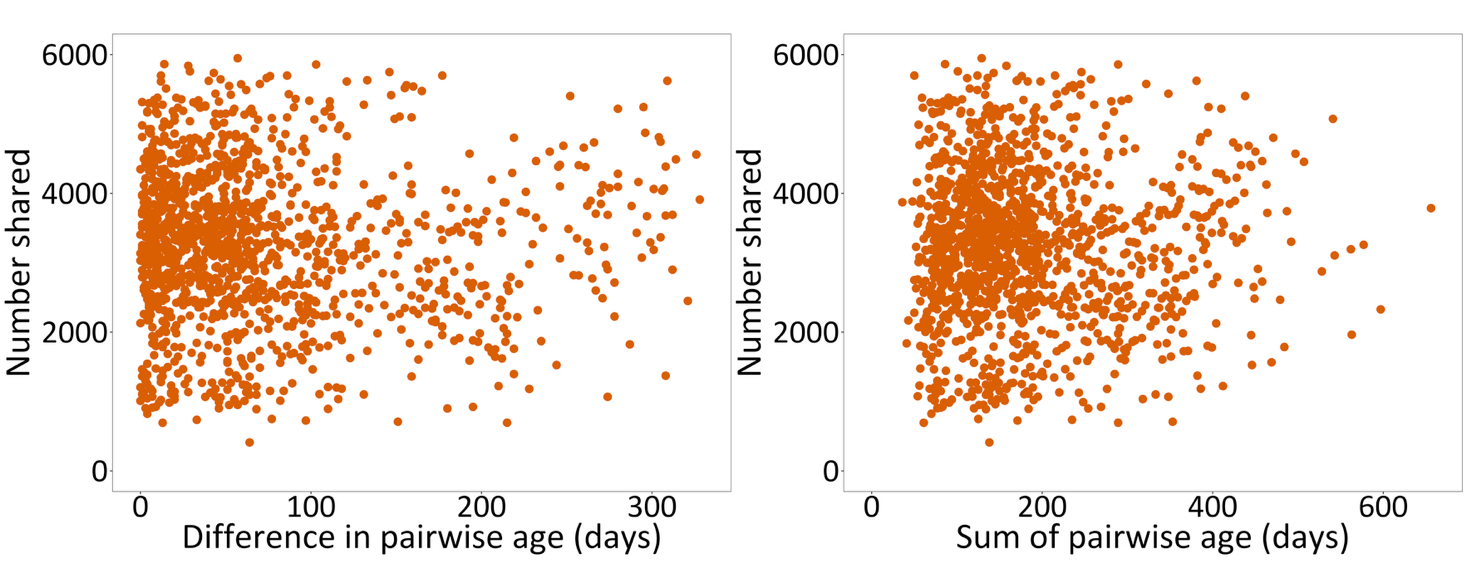


**B**


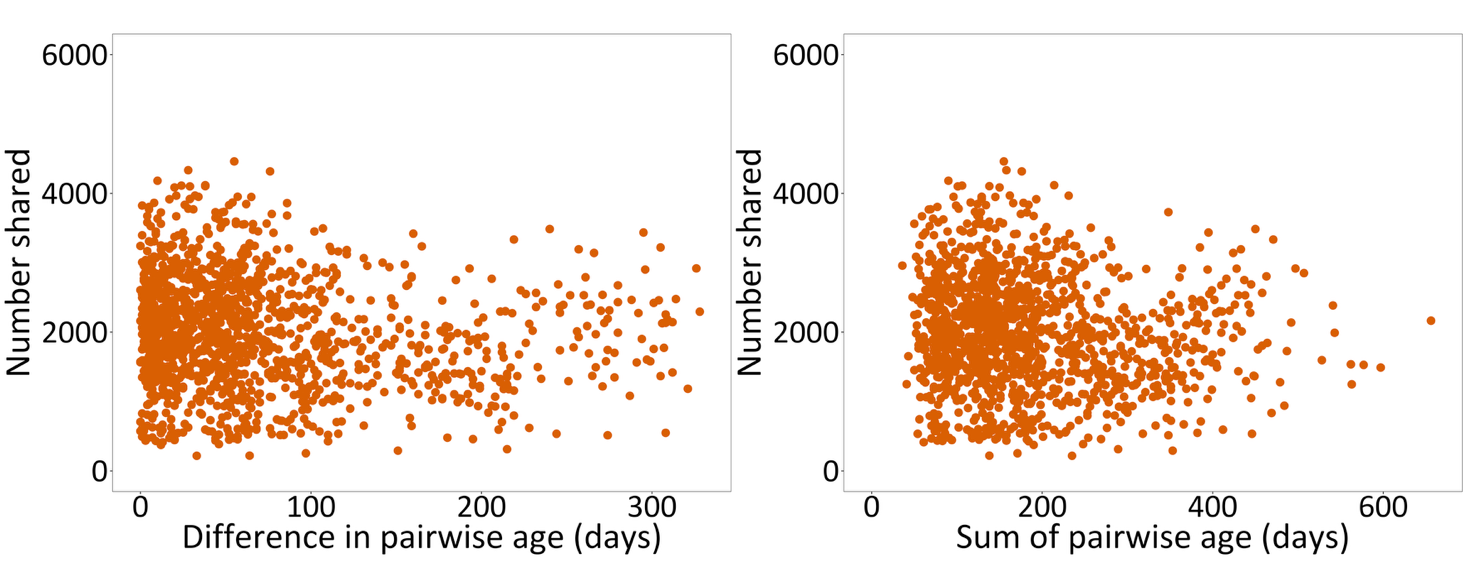


**C**


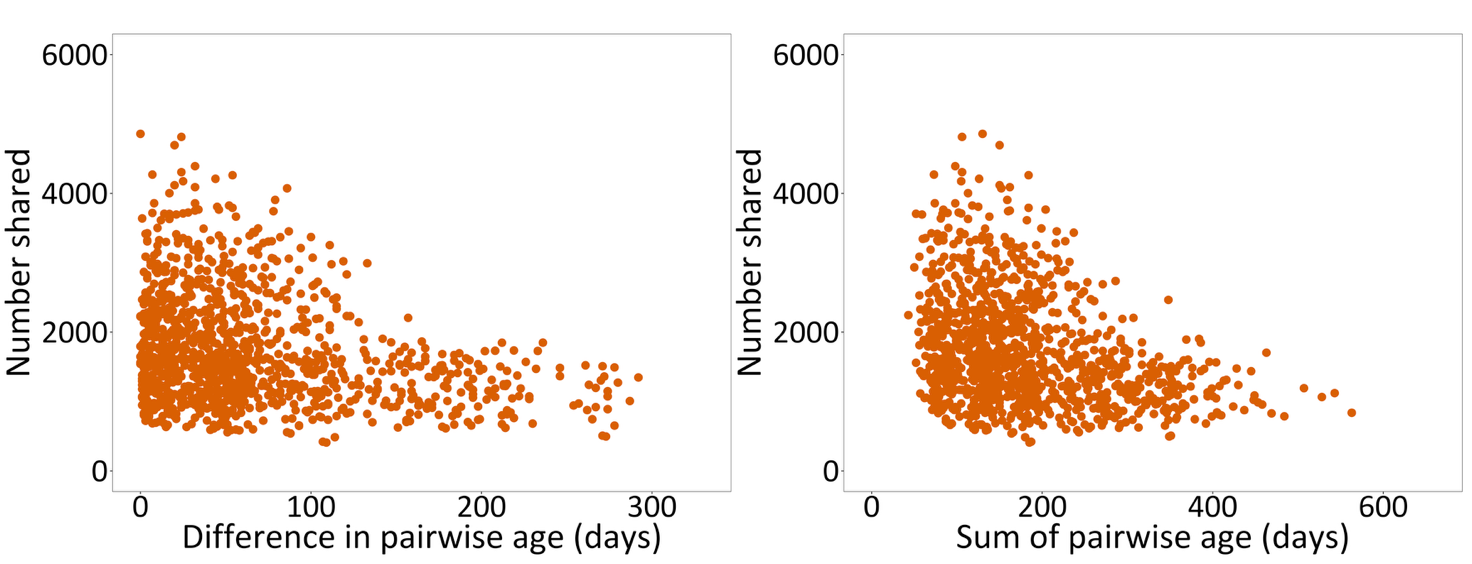


**Supplementary Material 11**. Analysis of the number of shared amino acid-defined TCR sequences and (A) pairwise difference in age and (B) pairwise sum in age of the mice, showing the slope estimates and 95 % confidence intervals (CI) for each receptor type (top table), and the P values for comparisons of the parameter estimates for the four receptor types (bottom table), with bold text showing p < 0.05. (C) Summary of pairwise shared amino acid-defined TCR sequences GLM. Estimates are shown relative to reference levels (CD4^+^ alpha, different sex pairs). Coefficients with p < 0.05 are in bold. GLM details were: n = 9,816; residual df = 9,803; null deviance = 1.14e10; residual deviance = 7.55e9; AIC = 160,917.

**A**

| **Receptor type** | **Estimate** | **95 % CI** |
| --- | --- | --- |
| **CD4^+^ alpha** | -0.541 | -1.23, 0.145 |
| **CD4^+^ beta** | -0.859 | -1.55, -0.173 |
| **CD8^+^ alpha** | -1.481 | -2.30, -0.665 |
| **CD8+ beta** | -1.368 | -2.18, -0.551 |

|  | **CD4^+^ alpha** | **CD4^+^ beta** | **CD8^+^ alpha** |
| --- | --- | --- | --- |
| **CD4^+^ beta** | 0.9182 |  |  |
| **CD8^+^ alpha** | 0.3091 | 0.6624 |  |
| **CD8^+^ beta** | 0.4258 | 0.7859 | 0.9975 |

**B**

| **Receptor type** | **Estimate** | **95 % CI** |
| --- | --- | --- |
| **CD4^+^ alpha** | 0.180 | -0.313, 0.6723 |
| **CD4^+^ beta** | -0.434 | -0.926, 0.0588 |
| **CD8^+^ alpha** | -1.392 | -1.965, -0.8193 |
| **CD8^+^ beta** | -2.110 | -2.683, -1.5368 |

|  | **CD4^+^ alpha** | **CD4^+^ beta** | **CD8^+^ alpha** |
| --- | --- | --- | --- |
| **CD4^+^ beta** | 0.3097 |  |  |
| **CD8^+^ alpha** | **0.0003** | 0.0620 |  |
| **CD8^+^ beta** | **<0.0001** | **0.0001** | 0.3049 |

**C**

| **Coefficient** | **Estimate** | **SE** | **t** | **p** |
| --- | --- | --- | --- | --- |
| **(Intercept)** | **3259.99** | **37.54** | **86.83** | **<0.001** |
| Age difference (days) | −0.54 | 0.35 | −1.55 | 0.122 |
| Sex (same *vs.* different) | 7.06 | 17.72 | 0.40 | 0.690 |
| Age sum (days) | 0.18 | 0.25 | 0.72 | 0.474 |
| **CD4^+^ beta (*vs.* CD4^+^ alpha)** | **−1120.06** | **51.76** | **−21.64** | **<0.001** |
| **CD8^+^ alpha (*vs.* CD4^+^ alpha)** | **−166.11** | **56.02** | **−2.97** | **0.003** |
| **CD8^+^ beta (*vs.* CD4^+^ alpha)** | **−1058.97** | **56.02** | **−18.90** | **<0.001** |
| Age difference *CD4^+^ beta | −0.32 | 0.50 | −0.64 | 0.521 |
| Age difference*CD8^+^ alpha | −0.94 | 0.54 | −1.73 | 0.084 |
| Age difference*CD8^+^ beta | −0.83 | 0.54 | −1.52 | 0.129 |
| Age sum*CD4^+^ beta | −0.61 | 0.36 | −1.73 | 0.084 |
| **Age sum*CD8^+^ alpha** | **−1.57** | **0.39** | **−4.08** | **<0.001** |
| **Age sum*CD8^+^ beta** | **−2.29** | **0.39** | **−5.94** | **<0.001** |
